# Supplementary material for: Highly active rubiscos discovered by systematic interrogation of natural sequence diversity
Source: EMBO J. 2020 Jun 5;39(18):e104081. doi: 10.15252/embj.2019104081 (PMC7507306; doi:10.15252/embj.2019104081)
Supplement: Supplementary file 1 — Appendix [file EMBJ-39-e104081-s001.pdf]

Appendix

Highly active rubiscos discovered by systematic interrogation of natural sequence diversity

Davidi *et al.* 2019
Correspondence: Ron Milo: ron.milo@weizmann.ac.il

All data and scripts used in this work are available at https://github.com/milo-lab/rubiscolympics.git

Table of Contents

Rubisco form assignment 2
Overexpression library construction 2
Expression of rubisco variants 3
Determining the effective extinction coefficient for NADH 4
Converting from decay of A340 absorption signal versus time to rubisco rate 5
Quality control filters for coupled assay results 5
Form-III pilot experiment 5
Oligomeric state determination 5
Michaelis -Menten kinetics via 14CO2-fixation assays 7
References 8

Rubisco form assignment

Form assignment was applied to identify all sequences of form-II and form-II/III rubiscos, which are the focus of this study. Further, it was used to remove form-IV rubiscos, which lack the carboxylation activity. For this, a total of 35,413 non-redundant rubisco homologs were clustered at 70% sequence identity. Centroid sequences (N=884) were then aligned and a phylogenetic tree was constructed (Figure S1). By mapping a curated set of 765 sequences to the tree from (Jaffe *et al*), we were able to infer the form of each centroid sequence. All sequences that clustered with a particular centroid were assigned the same rubisco form as their associated centroid.

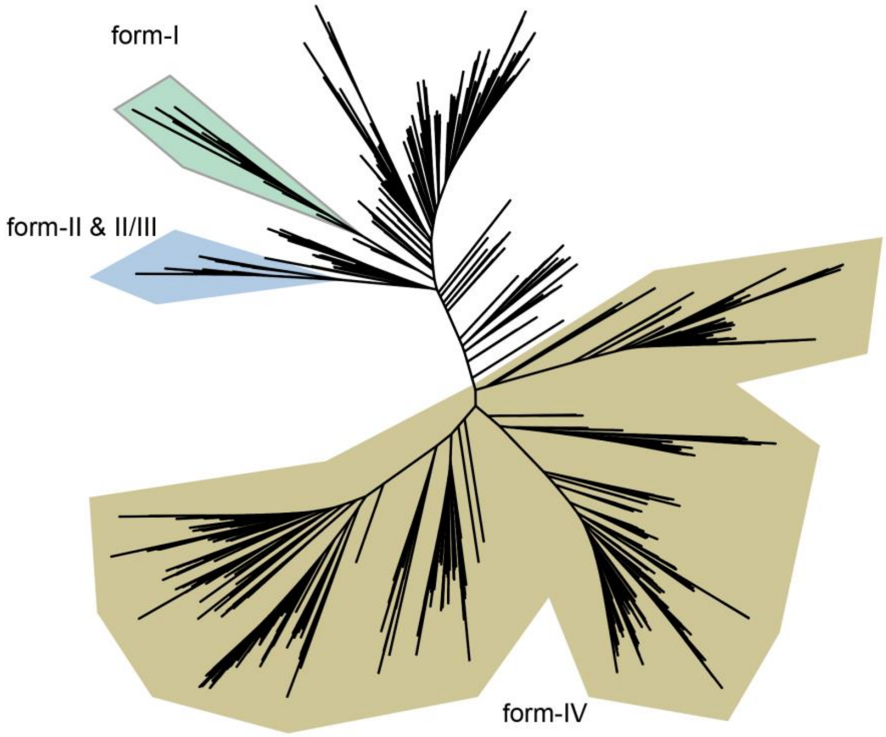

Figure S1: Maximum-likelihood phylogenetic tree of 884 centroid sequences that cover the sequence space of 35,413 rubiscos at 70% sequence identity. Form-IV rubiscos (light brown) appear as a distinct cluster, which was removed from the tree. The clusters of form-I and forms-II and -II/III are highlighted in green and blue, respectively. Other leaves of the tree either correspond to form-III variants or their form is unknown. For details on clustering and tree construction, see the Methods section.

Overexpression library construction

All 143 rubisco variants were cloned into a pET28 vector downstream to a modified His tag and SUMO fusion followed by a bdSENP1 protease cleavage site for scarless cleavage upon purification, as described in the Methods section and further in (Frey & Görlich, 2014). The first amino acid after the SUMO tag, i.e., the first amino acid of the coding sequence of rubisco, was maintained as indicated by the protein sequence database. Namely, if methionine was the first amino acid of a particular rubisco, it was used as the first amino acid downstream to the SUMO tag. If a rubisco sequence had a precursor sequence, the latter was removed manually to obtain the processed rubisco sequence. This was done by multiple sequence alignment to reference rubisco, as described in the Methods section. Each rubisco was, therefore, cloned in the following manner:

- pET28 backbone
- Modified His-tag
- SUMO tag

5’-
ctttaagaaggagatataccATGAGCAAGCATCACCATCATTCAGGCCATCACCATACCGGACACCACCATCATT
CAGGCAGTCATCACCATTCCGGATCTGCTGCGGGTGGCGAAGAAGATAAGAAACCGGCAGGTGGCGA
AGGTGGCGGTGCCCATATCAACCTGAAAGTGAAAGGTCAAGACGGCAACGAAGTCTTTTTCCGCATC
AAACGTTCTACCCAGCTGAAAAAGCTGATGAACGCATACTGTGACCGTCAGTCTGTAGACATGACCG
CAATTGCTTTCCTGTTTGATGGTCGTCGCCTGCGTGCGGAACAGACCCCGGATGAACTGGAGATGGAA
GATGGCGACGAAATCGACGCAATGCTTCATCAGACTGGTGGC - rubisco coding sequence -
tgagatccggctgctaacaa-3’

Expression of rubisco variants

Plasmids were transformed into *E. coli* BL21 cells and grown to mid-log (OD600 ≈ 0.6). Protein expression was then induced with the addition of 0.2 mM IPTG and cells were grown overnight at 16₀C. Upon cell harvest, the expression level of each variant was assessed by SDS-page of cell crude extracts (Figure S2). Only variants that showed a clear ≈63 kDa band were further purified. The 63 kDa corresponds to the tagged rubisco variants, as the average weight of a form-II rubisco monomer is 50 kDa and the His-SUMO tag, 13.4 kDa. Figure S2b shows a characteristic SDS-page of 12 rubisco variants after his-tag purification and cleavage. As can be appreciated, the purified proteins are relatively clean and the protein sizes decrease to 50 kDa, which reflects the tag’s removal. Notably, there is a high degree of variation in expression yield between variants, which may be due to, e.g., differences in expression intensity, protein solubility, and/or cleavage efficiency.

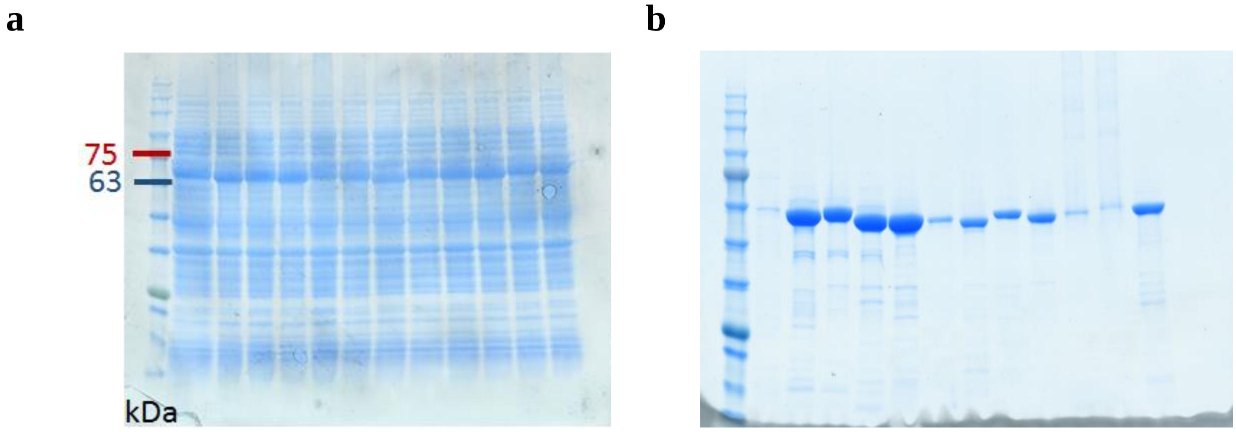

Figure S2: SDS-page analysis of rubisco variants before and after purification. **a.** A characteristic gel of crude lysates from E. coli cells expressing 12 different rubisco variants. Rubiscos are tagged and the total monomer size is ≈63 kDa. **b.** 12 rubisco variants after his-tag purification and SUMO cleavage. The size of the rubisco monomers is now ≈50 kDa; protein ladder is BLUeye prestained (GeneDirex hy-labs).

Figure S3 further illustrates the differences in purification yields across all variants, as determined by the Pierce BCA protein assay (Pierce™ BCA Protein Assay Kit; Thermo). Yield values are log-normal distributed, spanning three orders of magnitude. Because small amounts of rubisco were used in the carboxylation assay (50 nmol per assay), even variants with low expression yields were successfully assayed for their carboxylation activity.

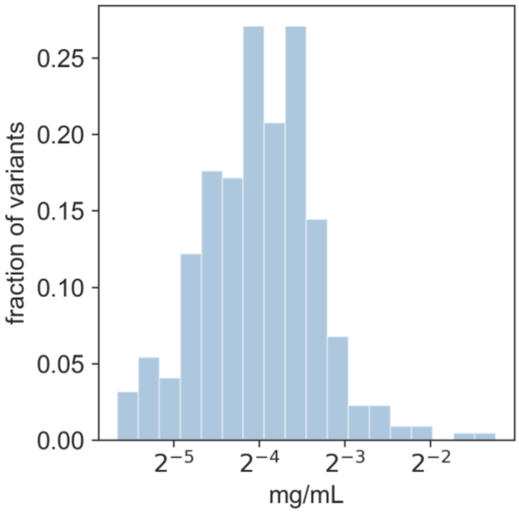

Figure S3: Distribution of protein purification yields for all variants expressed in this study; mg/mL was measured using BCA (Pierce™ BCA Protein Assay Kit; Thermo) according to the manufacturer's protocol.

Determining the effective extinction coefficient for NADH

The spectroscopic enzymatic assay for carboxylation was performed as described in Pierce *et al* with minor adaptations to suit the multiwell layout in a gas-controlled plate reader. Namely, instead of adding bicarbonate to the reaction mix, samples were incubated at 4% CO_2 for 15 minutes prior to the assay, and similarly, rubisco was activated by incubation at 4% CO_2 for 15 minutes, as opposed to incubation with 40 mM $NaHCO_3$.

For convenience, all assay components were mixed and distributed into a 96-multiwell plate, except for the rubisco enzyme and its substrate ribulose 1,5-bisphosphate (RuBP; see the Table S1). Only after the plate was incubated at 4% CO_2 and 0.4% O_2 for 15 minutes were the rubisco samples added to the wells. Activation was achieved by incubating the plate for a further 15 minutes at the same gas conditions. The assay started when RuBP was added to the wells, and immediately monitored for NADH oxidation in the gas-controlled plate reader.

Table S1: recipe for the spectroscopic coupled assay

Component	Assay concentration	Source
EPPS buffer pH 8.0	100 mM	Alfa Aesar (Cat # J61296)
MgCl2	20 mM	Sigma Aldrich (Cat# M2670-500G)
Dithiothreitol	0.5 mM	Bio Basic Canada inc. (Cat# DB0058)
ATP	2 mM	Sigma Aldrich (Cat # A3377-5G)
Phosphocreatine	10 mM	Sigma Aldrich (Cat # 27920-5G)
NADH	0.5 mM	Merck (Cat # 481913-1GM)
Carbonic anhydrase	0.1 mg/mL	Sigma Aldrich (Cat # C3934-100MG)
Creatine phosphokinase	20 U/mL	Sigma Aldrich (Cat# C3755-35KU)
Glyceraldehyde 3-phosphate dehydrogenase	20 U/mL	Sigma Aldrich (Cat# G2267-10KU)

3-Phosphoglyceric phosphokinase	20 U/mL	Sigma Aldrich (Cat # P7634-5KU)

Converting from decay of A340 absorption signal versus time to rubisco rate

The enzymatic assay used in our activity screen couples rubisco's carboxylation to NADH oxidation (Figure 3). The rate of NADH oxidation, which is equal to twice the rate of carboxylation, was monitored by the decay in absorbance at 340 nm. According to the Beer-Lambert law, the concentration of NADH, c, is given by:

$$c = e \cdot l \cdot A, \qquad \text{Eq. 1}$$

Where e is the molar extinction coefficient of NADH at 340 nm, l is the path length and A is the absorbance at 340 nm. Because we used a multiwell plastic plate instead of a standard 1 cm quartz cuvette, we empirically determined l in our system. For this, we serially diluted NADH into a multi-well plate containing all the assay components (except for RuBP, to avoid enzymatic NADH oxidation) and generated a calibration curve. The concentration of the NADH stock was determined spectroscopically using $e = 6.22\ L \cdot mol^{-1} \cdot cm^{-1}$and a 1 cm quartz cuvette. The slope of the fit line between the NADH concentration (x-axis) and absorbance (y-axis) is the effective conversion factor between A and c in our system and was found to be $\beta = 1.54$. Because the stoichiometry between NADH oxidation and the production of the carboxylation product, 3-phosphoglycerate, is 1:1, the carboxylation rate, v, is given by:

$$v = -\frac{\partial c}{\partial t} = -\beta \cdot A \,/\, 2. \qquad \text{Eq. 2}$$

Note that the negative sign is because the decay in signal reflects the production of carboxylation products. Further, the division by two is because each carboxylation reaction produces two 3-phosphoglycerate molecules.

Quality control filters for coupled assay results

To ensure accurate and quantitative measurement of carboxylation rates using our spectrophotometric assay, we applied several quality-control filters. First, we filtered out measurements for which less than three CABP concentrations were available. For example, if the concentration of rubisco is lower than 20nM, the relation between carboxylation rate and CABP concentrations, which is used to derive the specific activity of the enzyme (Figure 3), is only based on two data points and hence was excluded. Second, we removed measurements in which the CABP-based determination of rubisco concentrations was higher than 100nM. This implies that we were not able to obtain full inhibition of rubisco and thus were not able to accurately determine the noise level - that is decay in A340 that is not due to rubisco activity. Third, we filtered our measurements in which the rate at 0 nM CABP was <2 than the rate at 90 nM CABP, which indicates that the rate of rubisco is only twofold higher than the background rate. Overall, out of 143 variants tested, 78 passed all quality control filters.

Form-III pilot experiment

To test the feasibility of sampling the kinetic space of form-III rubiscos, a pilot experiment was conducted in which 18 distinct form-III rubiscos, sharing pairwise sequence identity of less than 80%, were *de-novo* synthesized and cloned into *E. coli*-compatible expression vectors (see Methods). Out of the 18 form-III variants synthesized, 14 variants were successfully expressed. Out of the successfully expressed variants, 8 showed low concentrations which did not allow us to determine their carboxylation rate using our spectrophotometric assay. Out of the remaining 6 variants, which were successfully assayed, 4 showed high background noise, and thus were filtered out from the analysis, leaving us with only 2 variants with measurable carboxylation rate.

Oligomeric state determination

For oligomerization state determination, 1 µg aliquots of enzymes (Figure S4) were analyzed by analytical gel filtration (Figure S5). Four rubiscos occupied a dimeric oligomeric state, including rubisco from *Gallionella sp.*

(OGS68397.1), the fastest reported to date. The rubisco from *S. caldicuralii* (WP_074200542.1) migrated as the hexamer, as previously observed for select form-II enzymes (Whitney *et al*, 2011). Notably, the rubisco from an ‘unknown organism’ (WP_052580230.1) was a dimer in the apo state (inactive state), which, as observed for *M.burtonii* rubisco (Gunn *et al*, 2017; Alonso *et al*, 2009), transitioned to a larger oligomer when bound to CABP (active state).

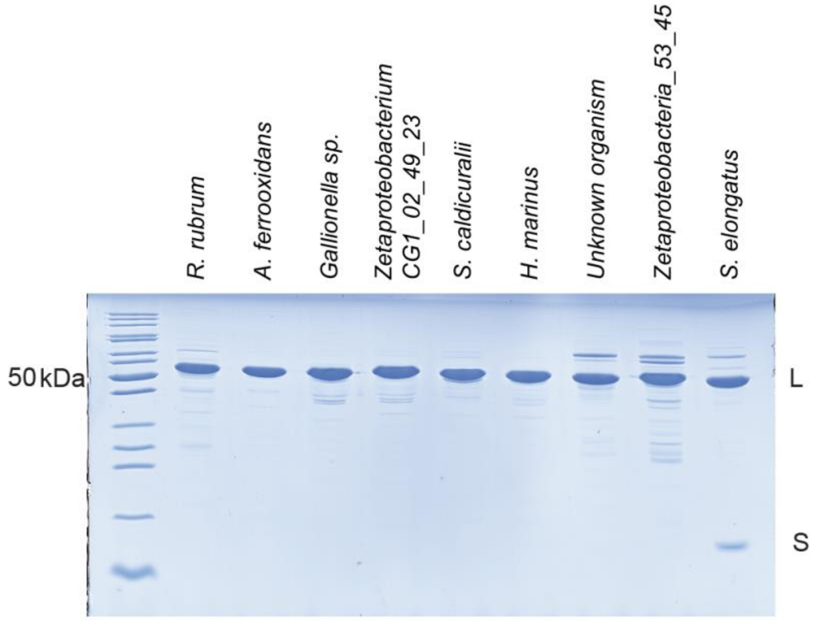

Figure S4: Purified enzymes were quantified using ^{14}C-CABP binding, and 1 µg aliquots of each rubisco were then analyzed using SDS-PAGE.

As a control, form-II rubisco from *R. rubrum* was used, which is the field’s gold standard form-II rubisco. Further, the form-I rubisco from *S. elongatus 6301*, which is composed of large and small subunits (Figure S4) and migrates as a L_8S_8 complex was used (Figure S5). Notably, the L_6 complex of rubisco from *A. ferrooxidans* (WP_012537012.1), which is one of the seven fast variants highlighted here, has previously been analyzed for its oligomeric state and was thus used as a control here (where, similarly, it migrated as a L_6 oligomer) (Tsai *et al*, 2015).

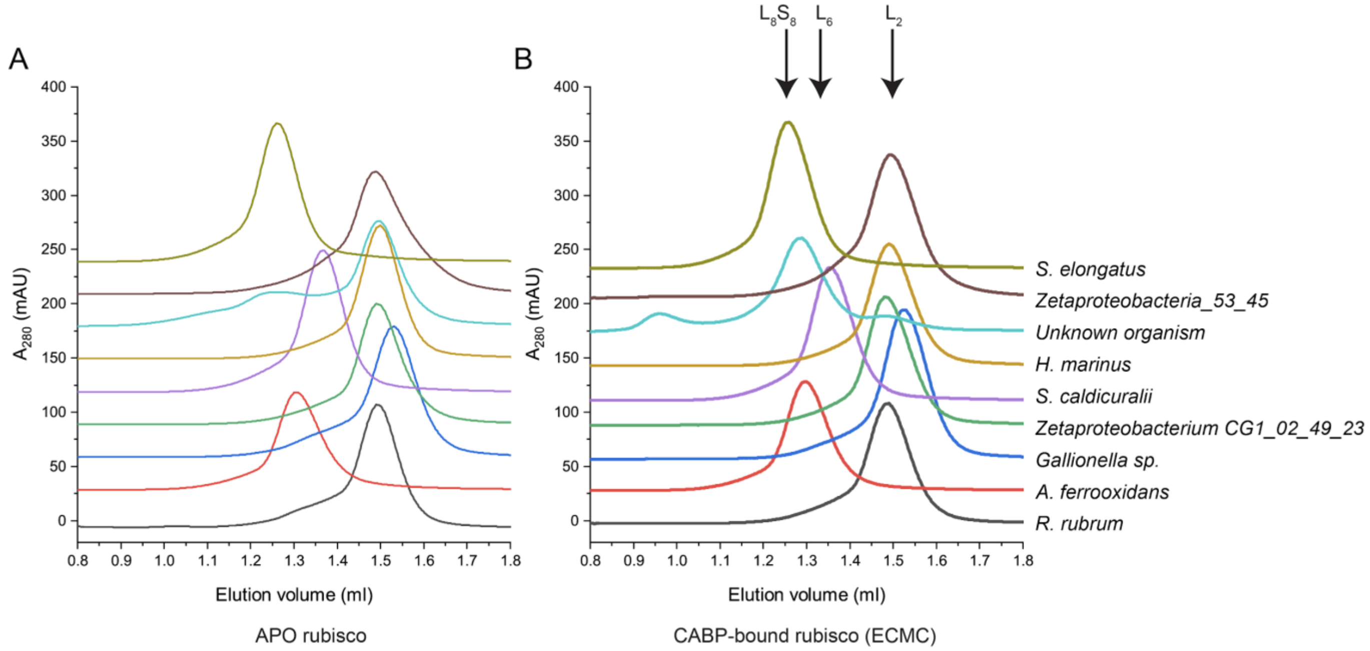

Figure S5: 30 µg aliquots of rubisco (as determined by 14C-CABP binding) in the APO (**A**) and CABP-bound state (**B**) were applied to a Superdex 200 PC3.2/30 column and eluted using 20 mM Tris-HCl, pH8.0, 50 mM NaCl. Previously determined oligomeric states are L8S8 (S. elongatus), L6 (*A. ferrooxidans*) and L2 (R. rubrum).

Michaelis -Menten kinetics via 14CO2-fixation assays

Purified enzymes were assayed for their absolute k_{cat} and K_M values for CO_2. Active site concentration was quantified by the ^{14}C-CABP binding assay (see Methods). Because rubisco from *S. elongatus* is the fastest reported until this study, we chose to remeasure it under the same assay conditions as our seven novel rubiscos. As a further control, form-II rubisco from *R. rubrum* was used.

In Figure S6 we show the Michaelis-Menten plots of the rubiscos assayed for their absolute carboxylation kinetics. Variants were measured in multiple replicates and data was fitted to a Michaelis-Menten model. The CO_2 concentrations used for the assay are compatible with the range previously reported measurements for *S. elongatus* (Andrews & Lorimer, 1985). We note that the maximum concentration, 500µM, is below 90% CO_2 saturation for most variants. This is because higher concentrations resulted in dampened carboxylation rates, probably due to substrate inhibition at high CO_2 concentrations.

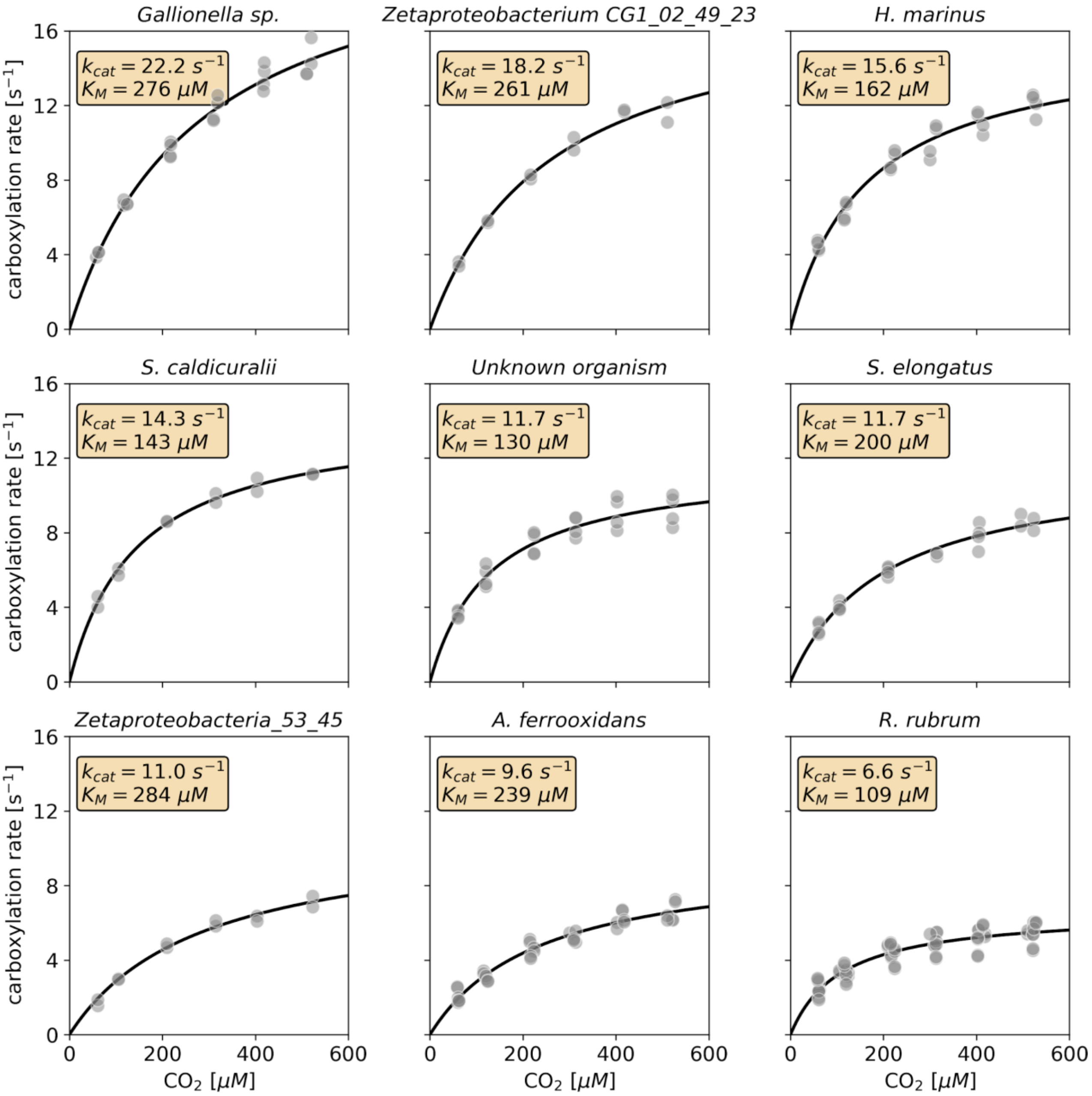

Figure S6: Michaelis-Menten plots of variants tested using ^{14}C fixation assay. Variants are sorted by their k_{cat} values (top left corner to bottom right corner) and include the R. rubrum rubisco and S. elongatus rubiscos as controls. Variants were measured in multiple replicates and data was fitted to a Michaelis-Menten model using python-scipy optimizer “curve_fit”; r values > 0.9.

References

Alonso H, Blayney MJ, Beck JL & Whitney SM (2009) Substrate-induced assembly of Methanococcoides burtonii D-ribulose-1,5-bisphosphate carboxylase/oxygenase dimers into decamers. *J. Biol. Chem.* **284:** 33876–33882

Andrews TJ & Lorimer GH (1985) Catalytic properties of a hybrid between cyanobacterial large subunits and higher plant small subunits of ribulose bisphosphate carboxylase-oxygenase. *J. Biol. Chem.* **260:** 4632–4636

Frey S & Görlich D (2014) Purification of protein complexes of defined subunit stoichiometry using a set of orthogonal, tag-cleaving proteases. *J. Chromatogr. A* **1337:** 106–115

Gunn LH, Valegård K & Andersson I (2017) A unique structural domain in Methanococcoides burtonii ribulose-1,5-bisphosphate carboxylase/oxygenase (Rubisco) acts as a small subunit mimic. *J. Biol. Chem.* **292:** 6838–6850

Jaffe AL, Castelle CJ, Dupont CL & Banfield JF Lateral gene transfer shapes the distribution of RuBisCO among Candidate Phyla Radiation bacteria and DPANN archaea. Available at: http://dx.doi.org/10.1101/386292

Tsai Y-CC, Lapina MC, Bhushan S & Mueller-Cajar O (2015) Identification and characterization of multiple rubisco activases in chemoautotrophic bacteria. *Nat. Commun.* **6:** 8883

Whitney SM, Houtz RL & Alonso H (2011) Advancing our understanding and capacity to engineer nature's CO2-sequestering enzyme, Rubisco. *Plant Physiol.* **155:** 27–35
